# Physical activity and change in quality of life during menopause -an 8-year follow-up study

**DOI:** 10.1186/1477-7525-10-8

**Published:** 2012-01-23

**Authors:** Jaana M Moilanen, Anna-Mari Aalto, Jani Raitanen, Elina Hemminki, Arja R Aro, Riitta Luoto

**Affiliations:** 1 School of Health Sciences, University of Tampere, Tampere, Finland; 2 Service system department, National Institute for Welfare and Health, Helsinki, Finland; 3 UKK Institute for Health Promotion, Tampere, Finland; 4 University of Southern Denmark, Esbjerg, Denmark; 5 Department of children, young people and families, National Institute for Welfare and Health, Helsinki, Finland

**Keywords:** Menopause, physical activity, longitudinal study

## Abstract

**Background and objectives:**

The aim of this study was to study the role of menopausal status and physical activity on quality of life.

**Methods:**

A total of 1,165 Finnish women aged 45-64 years from a national representative population-based study were followed up for 8 years. Study participants completed the Health 2000 study questionnaire and follow-up questionnaire in 2008. Ordinal logistic regression analysis was used to measure the effect of menopausal status on global quality of life (QoL). Other variables included in the analyses were age, education, change of physical activity as assessed with metabolic equivalents, change of weight and hormone therapy (HRT) use.

**Results:**

Peri- and postmenopausal women increased their physical activity (28% and 27%) during the eight-year follow up period slightly more often than premenopausal (18%) women (p = 0.070). Menopausal status was not significantly correlated with change of QoL. QoL of the most highly educated women was more likely to improve than among the less educated (e^b ^= 1.28, 95%CI 1.08 to 1.51 p = 0.002). Women whose physical activity increased or remained stable had greater chances for improved QoL than women whose physical activity decreased (e^b ^= 1.49, 95%CI 1.23 p < 0.001 to 1.80, e^b ^= 1.46, 95%CI 1.24 to 1.73 p < 0.001 respectively). Women whose weight remained stable during follow-up also improved their QoL compared to women who gained weight (e^b ^= 1.26, 95%CI 1.07 to 1.50 p > 0.01). Women who had never used HRT had 1.26 greater odds for improved QoL (95%CI 1.02 to 1.56 p = < 0.05).

**Conclusion:**

Improvement of global QoL is correlated with stable or increased physical activity, stable weight and high education, but not with change in menopausal status.

## Background

Women experience menopause between 40 and 58 years of age, the median age being 51 years [1]. Menopause is also a time of life with many symptoms and poor health status, which affect quality of life [2,3]. Menopause is also associated with a number of physical, psychological and social changes [4]. Many studies have found that the menopause is associated with deteriorating quality of life (QoL) [5]. Menopause may be accompanied by health problems with decreasing estrogen levels with symptoms such as hot flushes, night sweats and vaginal dryness. In a number of studies menopausal symptoms have been reported to be associated with quality of life indicators [6]. On the other hand there are also study results indicating that well-being is not associated with menopausal status per se but is associated with current health status [7].

Physical activity has been shown also to enhance quality of life among menopausal women [8,9] and some studies suggest that physical activity is associated with a decrease of hot flushes [10,11]. The effect of physical activity in decreasing hot flushes has been explained by β-endorphin theory. It is known that increase of hypothalamic β-endorphin production may stabilize thermoregulation known to be disturbed during menopausal hot flushes[12]. Physical activity may help in controlling body weight, which is associated with more frequent vasomotor symptom reporting [10,13]. It has been shown that weight gain in midlife is not specifically related to menopause but to aging [14-16], and gaining weight may impair quality of life [16,17]. The question whether menopausal transition could be considered as 'window of opportunity', i.e. whether there is any lifestyle modification during menopausal transition, is left open.

Our aim was to study the role of physical activity and menopausal status in change in quality of life among menopausal women.

## Methods

The baseline data come from a health examination study entitled Health 2000. This was carried out in Finland between 2000 and 2001 and has been described in detail elsewhere [18,19]. A nationally representative two-stage stratified cluster sample was drawn of adults aged 30 and over and living in mainland Finland. A total of 7,419 subjects (93% of the 7,977 subjects originally drawn from the population register) participated in one or more phases of the study. Data collection included an extensive home interview, three self-administered questionnaires and a clinical examination by a physician. The response rate for the home interview was 87.6% and for the first self-administered questionnaire 84.4% among the whole study population. The response rate among women aged 45-64 years at baseline was 86.6%.

In 2008 all respondents who were 37-56 years old in 2000 (45-64 years old in 2008) were sent a mailed questionnaire. After three reminders the overall response rate was 82.2% (n = 1,239). Of the respondents, 1 239 women who had responded both to the home interview and to the self-administered questionnaire were included into this study (n = 1,165). In the 8-year follow-up study most of the questions and indicators were similar to those in the baseline Health 2000 study (menopausal status, symptoms list, quality of life, general health, coping at work).

### Variables

Menopausal status and transition category

Women with a normal, regular menstrual cycle during the past 12 months were classified as premenopausal, women with an irregular menstrual cycle during the past 12 months as perimenopausal, and women whose last menstrual cycle had occurred more than 12 months ago as postmenopausal regardless whether HRT was used or not.

Three menopause transition categories were defined as: 1) premenopause at both baseline and follow-up (pre-pre), 2) transition from premenopause to peri- or postmenopause (pre-peri/post) and 3) perimenopause or postmenopause baseline and follow-up from perimenopause to postmenopause (peri-peri/post, post-post).

### Physical activity

Physical activity was measured by MET (Metabolic Equivalent) hours per week (continuous variable) in years 2000 and 2008. MET variables were categorized as less than 21 MET hours per week = low activity, 21-42 MET hours per week = moderate activity and more than 42 MET hours per week = high activity.

Change in physical activity was defined as MET per week in year 2000 minus MET per week in year 2008 and coded as decreased when the change was -1, -2 or -3, increased when the change was 1 or 2 and when there was no change it was 0.

Physical activity variable MET per week was measured in the 2000 questionnaire with the following questions: 1) How much time overall do you spend on heavy physical activity on those days when you exercise for at least 10 minutes?", 2) How much time overall do you spend on moderate physical activity on those days when you exercise for at least 10 minutes?", 3. How much time overall do you spend on brisk walking on those days you walk for at least 10 minutes?". In 2008 questionnaire physical activity variable MET per week was elicited with the questions: "How much time per week do you spend on" a) brisk walking and rapid movement from one place to another or for recreation, pleasure or fitness? b) do something that demands moderate physical effort, for example cycling, vacuuming, gardening or some other function that cause some breathlessness and increasing heart rate (do not count walking in this group)? c) do something that demands hard physical effort, for example, running, aerobics, heavy gardening or some other activity that causes heavy perspiration and rapid increase in heart rate. Response alternatives were 1 = not at all, 2 = less than 1/2 hour per week, 3 = one hour per week, 4 = 2-3 hours per week, 5 = 4 hours or more per week.

MET variable was developed for comparison of year 2000 and 2008 data concerning physical activity. First we calculated MET from year 2000 data: how long time did responders spend in physical activity (heavy, moderate and light) during each day (in minutes). Minutes were then converted to hours and multiplied by 7 (one week time). Thereafter physical activity was divided to five categories which were: 1 = not at all, 2 = less than 1/2 hour per week, 3 = one hour per week, 4 = 2-3 hours per week, 5 = 4 hours or more per week. Year 2008 data was already in this category format. MET variable was then calculated by multiplying all categories with specified coefficient to make all physical activity with different intensities comparable. Coefficients for different physical activities are shown in table 1 (table 1). Thereafter we calculated MET hours and divided them as 1) less than 21 MET hours per week = low activity, 2) 21-42 MET hours per week = moderate activity and 3) more than 42 MET hours per week = high activity [20].

**Table 1:** Coefficients for physical exercise

Time spent	Light physical exercise	Moderate physical exercise	Heavy physical exercise
1 = not at all	0	0	0
2 = less than 1/2 hour per week	+1.5	+2.5	+3.25
3 = one hour per week	+4.5	+7.5	+9.75
4 = 2-3 hours per week	+15.0	+25	+32.5
5 = 4 hours or more per week	+30.0	+50.0	+65.0

### Weight

Weight was measured in kilograms in both 2000 and 2008 surveys. Body mass index was defined as weight (kg)/height squared (m^2^). Change in (kg) weight was defined as weight in 2000 minus weight in 2008. Stable weight was defined as weight in 2008 between -5.0 and 5.0 kg of weight in 2000. Women who lost or gained more than 5.0 kg were classified respectively as weight losers and weight gainers.

### Quality of Life

Quality of life (QoL) was measured on the Ladder of Life scale modified by Andrew and Withey[21]. Respondents were asked to evaluate their QoL during the previous month. The scale was from 0 to 10 with 0 meaning worst possible quality of life and 10 meaning best possible quality of life. Responses were categorized as 0-4 (poor), 5-7 (moderate), 8 (good), 9-10 (excellent) [22]. Change in quality of life was defined as QoL in 2000 minus QoL of life in 2008 resulting in the categories -3, -2, -1 defined as deteriorated QoL, 0 defined as no change and 1 or 2 defined as improved QoL.

### Hormone therapy (HRT)

Hormone therapy (HRT) use was defined at baseline by last month of using HRT and at 8-year follow-up during year 2008 use during last six months. Categories in both baseline and follow-up surveys were similar- current, previous and never users. Current users were women who used HRT when they answering the questionnaire. Previous users had used HRT before but not now. Never users were those who did not report any use of HRT at baseline or follow-up, or the period between these survey timings.

### Statistical analysis

Baseline and follow-up characteristics were tabulated (mean and standard deviations) or as proportions and percentages. Differences between baseline and follow-up values were evaluated using McNemar's test. Changes in physical activity in different menopausal groups were tabulated with proportions and percentages. Differences between menopausal groups were tested using chi square test.

The effect of menopausal status and change in physical activity on quality of life changes was tested with ordinal regression analyses. The models included baseline QoL, age, education (primary, secondary and tertiary), weight change and use of hormone replacement therapy (HRT). We used ordinal logistic regression since the dependent variable (quality of life) was not normally distributed and was ordinal scale (-2 and -1 = declined, 0 = stable, 1 and 2 = improved) and thus did not meet the criteria for linear regression analyses. Quality of life change was used as dependent variable, menopausal status and change in physical activity (MET hours/week) as confounding factors (QoL at baseline, age, education, weight change as independent variables).

We used ordinal regression although testing parallel lines assumption showed that the general model did not greatly improve the fit. We also conducted a multinomial regression analysis because of this absence of assumptions and obtained the same results as in the ordinal regression. Ordinal regression is easier to interpret than multinomial regression analysis. Results of the ordinal model are interpreted in such a way that larger coefficients (> 1) indicate an association with larger scores, lower coefficients (< 1) association with lower scores, respectively.

The full model was adjusted for age, education, menopause status, change in physical activity and change in weight. All analyses were performed using the Statistical Package for the Social Sciences, version 15.0 statistical packages.

## Results

At baseline the mean age of the study sample was 47.0 years and in 2008 it was 56.0 years. The proportion of women reporting at least moderate physical activity (at least 21-42 MET hours) was higher at the follow-up as compared to baseline (p > 0.001). The proportion of women with overweight (33% to 34%) or obesity (24% to 23%) was stable over time. At baseline over half of the women (67%) were premenopausal and in 2008 only a fifth of women (19.9%). Among all women 38% reported excellent quality of life at baseline, but only 26% at 2008 follow-up. Proportion of women using HRT was higher at 2008 as compared to year 2000 (Table 2)

**Table 2:** Baseline characteristics of the cohort study: age, socioeconomic background and lifestyle (N = 1165)

	2000		2008		McNemar-test
	N	%	N	%	
**Age (SD)**	47.0 (5.2)		56.0 (5.2)		
**Education**					
Primary	309	26.5	-	-	
Secondary	381	32.7	-	-	
Tertiary	475	40.8	-	-	
**Quality of life^1)^**					< 0.001
0-4 (poor)	19	1.6	62	5.4	
5-7 (moderate)	291	25.0	337	29.2	
8 (good)	406	34.9	460	39.8	
9-10 (excellent)	446	38.4	297	25.7	
**MET hours/week^2)^**					
< 21	238	20.7	157	13.6	> 0.001
21-42	249	21.6	322	27.9	
> 42	665	57.7	677	58.6	
**BMI (kg/m2)^3)^**					0.068
< 25	521	45.2	478	42.3	
25-29.9	369	32.0	388	34.4	
> 30	263	22.8	263	23.3	
**Menopausal status^4)^**					> 0.001
Premenopausal	772	67.0	232	19.9	
Perimenopausal	82	7.1	170	14.6	
Postmenopausal	298	25.9	762	65.5	
**Hormone replacement therapy use^5)6)^**					> 0.001
current	267	23.0	425	36.9	
previous	128	11.0	183	15.9	
never	767	66.0	545	47.3	

1) missing 12

2) MET = metabolic equivalent hours, missing 47

3) missing 20

4) missing 14

5) missing 12

6) 2000: last month 2008:last six month

Women whose menopausal status changed from premenopause to perimenopause or postmenopause increased their physical activity (28% and 27%) during the 8-year follow-up more often than did premenopausal women (18%) and the differences were close to be significant (p = 0.070) (Table 3). There were no significant differences between the three groups in changes in QoL (p = 0.38) or weight change (p = 0.38). However, proportion of women whose quality of life deteriorated was higher among women in menopausal transition (41.5%) than compared to premenopausal women (34.5%) or postmenopausal women (34.9%). Proportion of weight gainers was highest among premenopausal women as compared to other groups (Table 3).

**Table 3:** Change of global QoL, physical activity and weight during 8-year follow-up by menopausal group

Changes	Pre-pre^a^)		Pre-peri/post^b)^		Peri-peri/post, post-post^c)^		p-value*
	(n = 229)		(n = 535)		(n = 762)		
	N	%	N	%	N	%	
**Quality of life^1)^**							
-2	19	8.3	40	7.5	27	7.2	0.376
-1	60	26.2	182	34.0	104	27.7	
0	103	45,0	221	41.3	177	47.2	
1	40	17.5	72	13.5	55	14.7	
2	7	3.1	20	3.7	12	3.2	
**Physical activity^2)^**							0.070
Decrease	52	22.6	109	20.6	79	21.2	
Stable	137	59.6	274	51.9	194	52.0	
Increase	41	17.8	145	27.5	100	26.8	
**Weight/kg^3)^**							0.379
Losers < 5 kg	21	9.2	64	12.0	49	13.1	
Stable ± 5 kg	157	68.6	377	70.6	257	68.9	
Gainers > 5 kg	51	22.3	93	17.4	67	18.0	

^1) ^missing 26

^2) ^missing 34

^3) ^missing 29

* tested with chi square test

^a) ^premenopause at both baseline and follow up

^b) ^transition from premenopause to peri- or postmenopause

^c) ^perimenopause or postmenopause both at baseline and follow-up; from perimenopause at baseline to postmenopause at follow-up

QoL of the most highly educated women had improved more than QoL of the least educated women (Table 4). Women whose physical activity increased or remained stable had improved QoL more often than women whose physical activity diminished. Women whose weight remained stable were more likely to have better QoL than women who gained weight during follow-up. Women who had never used HRT had better QoL than women who were current HRT. Women who were current HRT users had more deterioration in quality of life than women who had never used HRT (results not shown in the tables).

**Table 4:** Ordinal regression analysis for change in quality of life (dependent variable), results from a model including all covariates shown in the table

	N	e^β^	CI (95%)
**Age (years)**	1097	1.01	0.99 to 1.30
**Menopausal transition status**			
Pre-pre^a)^	225	1 (ref.)	
Pre-peri, post^b)^	513	0.93	0.77 o 1.14
Peri-peri/post.post-post^c)^	359	0.88	0.70 to 1.11
**Education**			
primary	284	1 (ref.)	
secondary	358	1.01	0.85 to 1.21
tertiary	455	1.28**	**1.08 to 1.51**
**QoL at baseline**			
poor	19	14.18	8.38 to 24.00
moderate	271	5.94	4.91 to 7.19
good	383	1.95	1.67 to 2.28
excellent	424	1 (ref.)	
**Physical activity change (MET/week)**			
decrease	232	**1**	
stable	590	1.46***	**1.24 to 1.73**
increase	275	1.49***	**1.23 to 1.80**
**Weight change**			
gainers	208	1	
stable	765	1.26**	**1.07 to 1.50**
losers	124	1.20	0.94 to 1.53
**HRT Use in baseline**			
Current	403	1	
Before	177	0.73	0.73 to 1.18
Never	517	1.26*	**1.02 to 1.56**
			
R^2 ^(Nagelkerke)	0.340		

Interpretation of results: if eβ > 1, the odds of higher QoL are greater, whereas when eβ < 1 low QoL is more probable.

*p < 0.05

**p < 0.01

***p < 0.001

^a) ^premenopause at both baseline and follow up

^b) ^transition from premenopause to peri- or postmenopause

^c) ^perimenopause or postmenopause both at baseline and follow-up; from perimenopause at baseline to postmenopause at follow-up

## Discussion

The aim of this study was to assess the relationships between changes in quality of life, menopausal status and physical activity. Change in global quality of life is more associated with change in physical activity than change in menopausal status. Similar findings have been reported in other studies [23]. However, women whose physical activity or weight remained the same, physical activity increased or women who were the most highly educated, had improved QoL over time. Mishra et al. [24] in their longitudinal study with 2 years of follow-up found that certain domains of QoL decline with aging and physical aspects of general health and well-being measured by SF-36 scale declined during the menopausal transition. Women who were perimenopausal for at least a year reported greater decline in their physical health and psychosomatic domains than did premenopausal women [24].

Peri- and postmenopausal women increased their physical activity during the 8-year follow-up compared to premenopausal women Physical activity has been reported to decrease with age [25], but in our study it seems that women in menopausal transition changed their behaviour in another direction. Increased motivation for lifestyle modification during menopausal transition could explain this increasing physical activity.

Elavsky et al. [26] in their longitudinal study found that physical activity improves physical self-worth and positive affect and that the improvements in affect lead to improvements in QoL. In our study those women who decreased their physical activity had deterioration in QoL than did women whose physical activity remained stable. Women who increased their physical activity improved their QoL. In the study by Elavsky et al. [26] increase in physical activity mediated positive affect and therefore had an effect on QoL. Some other studies claim that physical activity alleviates menopausal symptoms (hot flushes) and so improves QoL [11,27]. It is hypothesized that endorphin concentration in the hypothalamus decreases and oestrogen production declines, facilitating the release of norepinephrine and serotonin. Exercise may have similar ameliorating effects on vasomotor symptoms by increasing the presence of hypothalamic and peripheral β-endorphin production. Through these mechanisms, exercise may help to stabilize the thermoregulatory centre and diminish the risk of hot flushes [28]. The relationship between physical activity and QoL during the menopause is complex and may involve a number of alternative mechanisms, physiological or psychological or both.

Women who gained weight were more likely to report deterioration in QoL. This is consistent with other studies [23,16]. The eight-year follow-up study by Dennerstein et al.[23] found that increase of body mass index was associated with decline in self-rated health. Whether this is because of their knowledge of the relationship between body fatness and chronic disease or whether it reflects a problem with body image is unknown. In the study by Sammel et al. [16] the major predictors of weight gain among menopausal women were quality of life and other psychological factors including depressed mood and anxiety. One might speculate some causality between these factors; did weight gain lead to decline in QoL or did poorer QoL lead to weight gain? The Study of Women's Health Across the Nation (SWAN) [29], which is a multiethnic cohort study, found that women who had gained weight during the study period reported more vasomotor symptoms (hot flushes) than women whose weight remained stable and hence also poorer QoL.

However, the question of association between obesity and vasomotor symptoms is contradictory. Obese postmenopausal women have an increased peripheral conversion of androstenedione to estrone in adipose tissue compared to normal weight women, might be associated with fewer hot flushes in overweight women. On the other hand, lean women have more hot flushes at the time of menopause [30] and they have been shown to use HRT more frequently [31], although opposite results also exist [32]. Stadberg et al [33] found that a higher BMI was correlated with a higher climacteric symptom score. They thought that possible reason maybe that overweight women sweat more often because of their extra weight load and obesity is also associated with less exercise and poorer general health. Overweight could also be viewed as a lifestyle factor with less concern about health and lower self-esteem [34].

It was a limitation of our study that information on QoL was elicited only in one straight question, not different dimensions of quality of life, as for example the SF-36 Health Survey questionnaire. In SF-36 scale there are 36 items assessing eight dimensions of quality of life. The global QoL scale is self-anchoring because ratings are made relative to each person's conception of her best or worst QoL. Nor could we use symptoms questions in a longitudinal perspective because the questions in the baseline and follow-up studies were different and thus not comparable.

The main strengths of our study were the size and the fact that it was longitudinal study on Finnish female population with a high response rate. The longitudinal nature of the study meant that it was possible to thoroughly investigate change in physical activity and other related factors during the menopausal transition between two time points.

## Conclusion

Menopausal transition was not significantly correlated with change in global QoL during 8-year follow-up. However, women who increased their physical activity, had stable weight or were most highly educated had improved QoL. Our study pinpoints the importance of physical activity increase during menopausal transition and also supports the hypothesis that menopause may be a window of opportunity, since it may induce lifestyle modification.

## Competing interests

The authors declare that they have no competing interests.

## Authors' contributions

RL and JMM originated the idea for study. RL, JMM, AMA, ARA and EH planned the study questions and analysis. JR and JMM were responsible for statistical analysis. JMM prepared the first version of the manuscript. All authors (RL, AMA, EH, ARA, JMM and JR) participated in drafting of manuscript and approved the final version.
